# Genome-wide scan reveals genetic divergence and diverse adaptive selection in Chinese local cattle

**DOI:** 10.1186/s12864-019-5822-y

**Published:** 2019-06-14

**Authors:** Lingyang Xu, Liu Yang, Bo Zhu, Wengang Zhang, Zezhao Wang, Yan Chen, Lupei Zhang, Xue Gao, Huijiang Gao, George E. Liu, Junya Li

**Affiliations:** 1 Innovation Team of Cattle Genetic Breeding, Institute of Animal Sciences, Chinese Academy of Agricultural Sciences, Beijing, China; 2 Farm Animal Genetic Resources Exploration and Innovation Key Laboratory of Sichuan Province, Sichuan Agricultural University, Chengdu, 611130 China; 3 Animal Genomics and Improvement Laboratory, United States Department of Agriculture-Agricultural Research Service, Beltsville, MD 20705 USA

**Keywords:** Chinese local cattle, Population structure, Selection signatures, Haplotype diversity, Adaptation

## Abstract

**Background:**

Understanding the population structure and genetic bases of well-adapted cattle breeds to local environments is one of the most essential tasks to develop appropriate genetic improvement programs.

**Results:**

We performed a comprehensive study to investigate the population structure, divergence and selection signatures at genome-wide level in diverse Chinese local cattle using Bovine HD SNPs array, including two breeds from North China, one breed from Northwest China, three breeds from Southwest China and two breeds from South China. Population genetic analyses revealed the genetic structures of these populations were mostly related to the geographic locations. Notably, we detected 294 and 1263 candidate regions under selection using the *di* and iHS approaches, respectively. A series of group-specific and breed-specific candidate genes were identified, which are involved in immune response, sexual maturation, stature related, birth and bone weight, embryonic development, coat colors and adaptation. Furthermore, haplotype diversity and network pattern for candidate genes, including *LPGAT1, LCORL, PPP1R8, RXFP2* and *FANCA*, suggest that these genes have been under differential selection pressures in various environmental conditions.

**Conclusions:**

Our results shed insights into diverse selection during breed formation in Chinese local cattle. These findings may promote the application of genome-assisted breeding for well-adapted local breeds with economic and ecological importance.

**Electronic supplementary material:**

The online version of this article (10.1186/s12864-019-5822-y) contains supplementary material, which is available to authorized users.

## Background

Cattle, as an important domesticated farm animal, is raised for many purposes, including meat, dairy, leather, and labor. Most of the modern cattle can be categorized into two subspecies: taurine cattle (humpless) and indicine cattle (humped) with dramatic phenotypic differences. The process of domestication was estimated to start approximately 11,000 years ago separately in the Fertile Crescent for *Bos t. taurus* and the Indian Subcontinent for *Bos t. indicus* [1, 2]. Selection (natural and human-imposed) and nonselective forces (introgression and the demographic events) drove the changes of cattle genome. Their combined effects have generated remarkable phenotypic diversity and genetic adaptation to local environment in global cattle populations [3, 4].

China has rich cattle genetic resources, and these breeds can be divided into different groups based on their locations, morphologies and sex chromosome polymorphisms [5–7]. Previous genetic analyses using mtDNA and Y microsatellite (Y-STR) data indicated that Chinese cattle breeds are influenced by the two above subspecies, with cattle in the north and northeast China are primarily of *Bos t. taurus* ancestry and cattle in southern China are predominantly of *Bos t. indicus* [5–11]. In contrast, cattle lived between North and South regions are mostly of *Bos t. taurus*, *Bos t. indicus* hybrids [12]. Modern farm animals are a result of selective breeding for many traits of economic and adaptive importance since domestication. Accurate identification of genomic regions involved with selection signatures is essential for illustrating the mechanism underling genetic variation contributing to phenotypic diversity [13]. Therefore, Chinese cattle represent geographically and biologically diverse cattle breeds and can offer valuable genetic resources for investigating the population structure, admixture and selection signatures of the cattle genome. Recently, high density SNP array and next-generation sequencing have markedly expedited the studies of the genetic bases underline important economic and adaptive traits in domestic animals. These technologies and related approaches have been utilized to explore process of domestication, and identify natural and artificial selection signatures in the divergent populations of dogs [14, 15], pigs [16], chickens [17] and horses [18].

Selection signature analyses can help to identify numerous candidate genes associated with production traits in domesticated animals. For example, using selection sweep analysis, Rubin et al. revealed that several genes (*IGF1*, *TSHR*, *PMCH,* etc) are associated with growth and metabolic regulation in chicken, and another study demonstrated that *NR6A1*, *PLAG1*, and *LCORL* are associated with morphological changes in pigs during domestication [16, 17]. Also, a recent genome-wide investigation of world-wide sheep breeds suggested a list of candidate genes under recent selection that were related to body size, growth, skeletal morphology, coat pigmentation, reproduction and adaptations to local climates [19–22]. In dairy and beef cattle, many studies have been conducted to explore the selection signatures in commercial breeding populations and revealed a list of candidate genes and SNPs, which were related to milk and meat production, fertility, disease resistance traits [23–32]. Increasing productivity and efficiency will be important, but maintenance of genetic diversity will also be crucial. To better understand cattle genome and its function in adaptation, many other studies have been conducted to investigate the genome selection signatures, and obtain a large numbers of genes which play a pivotal role in adaptation during domestication process [3, 4, 30, 33, 34].

Many studies have explored the population structure and selection signatures in world-wide and local cattle populations [3, 4, 25–27, 30, 33–36]. The high density SNP array has previously been shown to improve the detection of positive selection and to reduce false discoveries [4, 25, 37, 38]. To comprehensively uncover genetic differentiation in cattle genome, we performed a comprehensive study using high density SNP array to investigate the genetic structure, divergence and selection signatures in diverse Chinese cattle across a broad latitudinal range. The primary aims of current study were to 1) investigate the population structure within Chinese local cattle by sampling eight populations across a broad latitudinal range; 2) identify the genomic selection signatures involved in natural and artificial selection for local cattle population; and 3) explore the potential genetic bases underline adaptive traits in diverse range of environments.

## Methods

### Sample selection and genotyping

We genotyped a total of 179 samples using Illumina BovineHD SNPs array (contains a total of 777,962 SNPs) from eight cattle breeds including Yanhuang cattle (YHC), Menggu cattle (MGC), Caidamu cattle (CDM), Liangshan cattle (LSC), Pingwu cattle (PWC), Zhaotong cattle (ZTC), Wenshan cattle (WSC) and Nandan cattle (NDC). The full name, associated abbreviation for each breed and additional sampling information were presented in Additional file 1: Table S1. All samples were divided into four groups (North, Northwest, Southwest and South) based on their geographical distribution. We utilized PLINK v1.07 and custom R scripts for file conversion and SNPs quality control [39]. All samples showed a genotyping call rate of more than 95%. Closely related individuals (the PI-HAT value was greater than 0.25) were removed. Only autosomal SNPs were used for subsequently analyses. Also, the SNPs were filtered with the criterion of geno < 0.1 and MAF < 0.05.

### Genetic diversity, heterozygosity and current effective population size

Analyses of genetic diversity and effective population size were conducted for eight diverse breeds in China. These include two breeds (YHC, MGC) from Northern China, one breed from Northwest China (CDM), two breed from Southwest China (LSC and PWC), three breeds from South China (ZTC, WSC and NDC) (Additional file 1: Table S1 lists the breed names, breed codes, and their sampling location information including geographic origin, latitude, longitude and altitude). Genetic diversity was estimated using the distributions of minor allele frequency (MAF) and the average MAF across breeds. The observed heterozygosity (Ho) was estimated using PLINK v1.07 with option –hardy. *Ne* was calculated using the software SNeP v1.1 as described before [40]. The method inferred *Ne* based on LD against past *t* generations, where *t* = 1/2*c* and *c* is the distance between SNPs in Morgans (100 Mb = 1 Morgan was assumed) [41].

### Population structure and phylogenetic analysis

We conducted Multidimensional scaling (MDS) analysis using 78,865 SNPs after LD filter (r^2^ > 0.2). Pairwise genome-wide identity-by-state (IBS) pairwise distances were estimated for samples cluster using PLINK v1.07 (−mds -plot 4). Population admixture was examined using 12,478 SNPs after strict LD-based filter (r^2^ > 0.02) in STRUCTURE 2.3.4 [42, 43]. Each process was implemented using 10,000 replicates and 10,000 burn-in cycles under admixture and correlated allele frequencies models. We estimated the genetic distance (D) between pairwise combination of individuals using approaches implemented in PLINK v1.07 [39], where D = 1-(IBS2 + 0.5IBS1)/*N*, and IBS1 and IBS2 were the number of loci that share either one or two alleles identical by state, respectively and the *N* was the number of loci [44]. We next built Neighbor-joining phylogenetic tree using PHYLIP v3.69. The phylogenetic tree was visualized with Figtree 1.3.1, as reported before [4]. Patterns of splits and mixtures history of the populations were evaluated using TreeMix v1.13 [45]. To further test for evidence of admixture across populations, ancestry graph, three-population (*f3*) and four-population (*f4*) tests implemented in TreeMix were utilized to examine the presence of admixture [45, 46].

### Genome-wide LD estimation

We estimated LD across each breed as our previously reported [4]. In brief, pairwise LD (r^2^) for all retained SNPs were estimated using the PLINK “-ld” option with the default window size of 1 Mb, and the “–ld-window-r2” was set to 0 to generate all pairwise results. The LD decay was further evaluated and fitted against genomic distance using smooth.spline function in R (v3.2.4).

### Identification of selection signatures

To explore differences among groups from different geographic zones, we estimated the *di* statistics based on the unbiased estimates of pairwise *F*_*ST*_. We then combined individual pairwise *F*_*ST*_ between groups (North, Northwest, Southwest and South) and averaged across all SNPs to obtain the standardized summary *di* statistic [15]. Then, we estimated the *di* value for each group in a nonoverlapping sliding window with 50 neighboring SNPs. Selection regions were defined as those ranking in the top 1% windows with highest average *di* values [4, 15, 47].

To infer recent selection sweeps, we further utilized the Integrated Haplotype score (iHS) method to measure structure of haplotype, which essentially reflected unusually long haplotypes across genome [47]. iHS was estimated using *selscan* with default settings (except for the maximum gap was set to 800,000) [48]. To identify genomic regions with signals of selection, we considered 100-kb nonoverlapping windows, the density of signal in each region was evaluated according to the proportion of SNPs with |iHS| > 2. Finally, we defined candidate regions under selection as those top 1% in the empirical distribution (regions with < 10 SNPs were removed) [47].

### Gene annotation of selection regions

Based on the bovine UMD3.1 reference genome assembly, we retrieved genes within the intervals spanning the candidate regions from the UCSC genome browser. The gene enrichment analyses were performed using Database for Annotation, Visualization and Integrated Discovery (DAVID) functional annotation tools [49]. Only clusters with enrichment scores more than 1 (*P*-value < 0.05 after Bonferroni multiple test) were considered.

### Haplotype diversity and haplotype network analysis of candidate genes

To explore the diversity of haplotypes and evolutionary patterns of candidate genes cross groups/breeds, we first extracted SNP genotypes in each gene as unphased information, then the haplotypes were constructed and their frequencies were estimated using PHASE v2.1 [50]. To obtain reliable results, we also performed inference with 10,000 iterations and 10,000 burn-ins, and the final results were obtained using option -X 100 in PHASE v2.1. Haplotype networks was constructed and evaluated for genes (*LCORL*, *LPGAT1*, *PPP1R8*, *RXFP2* and *FANCA*) using the functions *haplotype* and *haploNet* from the *pegas* package [51].

## Results

### Genetic diversity, heterozygosity and current effective population size

After quality control, we obtained a total of 643,772 autosome SNPs for subsequent analyses. The call rates of SNP were generally high across samples (call rate > 0.95). We observed the distributions of minor allele frequency (MAF) display distinct patterns across breeds, which may reflect the diverse population history, genetic structure and geographic origin of breed formation. We also observed the average MAF ranged from 0.16 to 0.26 (Additional file 1: Table S1). Among them, WSC and NDC from southern China, which are indicine-derived population, had low MAF compared to taurine-derived populations in north and northwest China. To investigate diversity across our data set, we further calculated observed heterozygosity (*Ho*) within breeds (Additional file 1: Table S1). We observed the average *Ho* ranged from 0.22 to 0.35, and animals from South China displayed the lowest heterozygosity. In addition, we found the estimated *Ne* at 13 generations ago in Chinese naive cattle ranged from 85 to 132, which were consistent with previous current *Ne* estimation in the majority of breeds of domestic cattle (a current *Ne* of 150 or less) (Additional file 4: Figure S1).

### Population structure, admixture and phylogenetic analysis

**Fig. 1:**
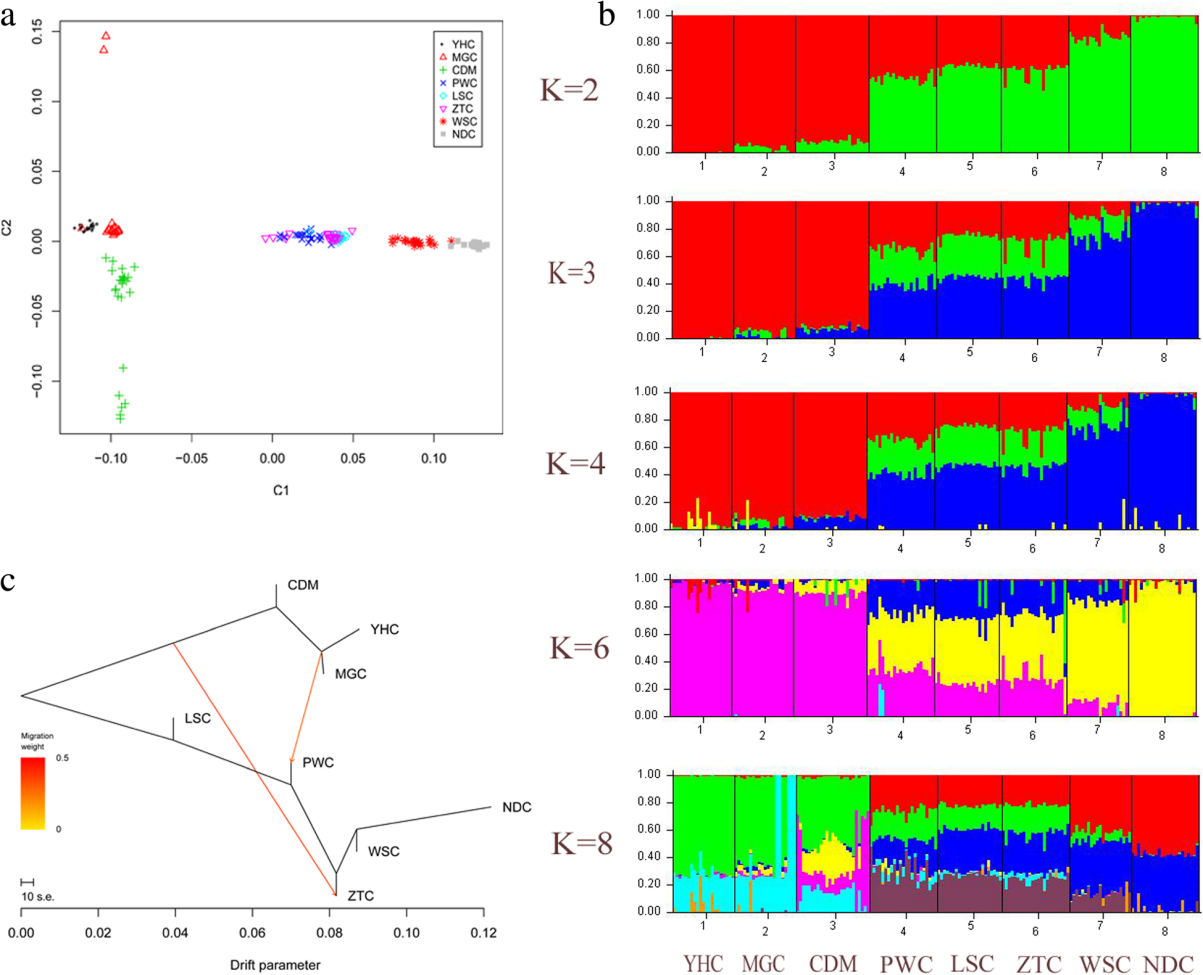
Population genetic analyses of eight diverse breeds. **a**. MDS analysis results of 179 individuals. Individuals are plotted according to their coordinates on the first two components. **b**. Clustering of 179 individuals based on LD filtered SNPs when K = 2 to 8. Individuals were shown as a thin vertical line colored in proportion to their estimated ancestry. **c.** *Maximum* likelihood tree inferred from 8 populations with two migration edges. The scale bar depicts ten times the average standard error of the estimated entries in the sample covariance matrix

All samples were divided into four groups including North, Northwest, Southwest and South. Seven of them are representative of Chinese local cattle, while YHC shows nearly 75%~ 80% of genetic pool contributed by Yanbian cattle in Northern China, with the remaining contribution from Limousin cattle of France. MDS analysis presented a clear genetic structure among the eight breeds from different geographical areas. Our result revealed the first dimension separates the North breeds (primarily originate from taurine) and South breeds (primarily originate from indicine), while the second dimension separates the breeds from highland (CDM) and breed from lowland (MGC) (Fig. 1a). The separated clusters indicated that the studied population was reasonable to investigate the genomic characteristics of these breeds. To identify admixture level, we performed admixture analysis using 12,478 LD-filtered SNPs with the K (number of clusters) varied from 2 to 8. When K = 2 or 3, the clustering pattern implied the remarkable division of cattle from North and South China. When K = 4, we observed the three breeds LSC, PWC and ZTC displayed expected levels of admixture, agreeing with their breed history (Fig. 1b). While for K = 6 to 8, we did not found any additional breed was separated.

We constructed a neighbor-joining tree using pairwise nucleotide genetic distances as described before [39, 52]. We observed animals from the same breed almost clustered together, while slight difference was found in the internal branches within a breed (Additional file 5: Figure S2). The pattern was consistent with previous report that modern breeds in China were derived from both taurine and indicine, and the formation of them may happen in a short period of time [7, 9]. We next estimated patterns of LD decay among them, and we found the average r^2^ generally decayed rapidly against distances as previously described [3, 4, 24, 53]. The pattern of LD and the decay trend differed between breeds and could be distinguished according to the breed type (Additional file 6: Figure S3). In agreement with previous studies, we also observed indicine breeds (South and Southwest) had shorter LD compared to taurine (North and Northwest) and the composite (hybrid) breeds [3, 53].

To further test for evidences of admixture across populations, we assessed the population history with admixture events using maximum likelihood approaches implemented in TreeMix. Our findings also confirmed several features already detected by STRUCTURE (Fig. 1c). As migration edges showed introgression between groups, we colored migration edges according to percent ancestry received from the donor population. Vectors 1–2 denoted gene flow from domestic North and Northwest into South and Southwest groups, mirroring the K = 2 results from STRUCTURE (Fig. 1b). The first vector indicated gene flow from the Northern group (MGC, YHC and CDM) to ZTC (Fig. 1c). The second vector connected PWC to the North group (MGC and YHC), indicating gene flow from MGC and YHC to PWC. To evaluate the presence of admixture, we further utilized the threepop and fourpop program implemented in the TreeMix v1.13 package to compute *f3* and *f4* statistics. The significant negative *f3* statistic values indicated that population A was admixed population and related to B and C. In this study, *f3* test results confirmed the admixture of PWC and ZTC. Our results suggested the three most extreme *f3* scores (− 34.22, − 31.96 and − 32.22) to the geographically proximate populations of YHC|NDC, MGC|NDC and CDM|NDC as sources of admixture for PWC. The similar introgression pattern with extreme *f3* scores (− 34.41, − 33.09 and − 32.44) was observed for ZTC from three populations (MGC, YHC and CDM). In addition, we did not observe significant result for the *f4* test.

### Candidate selection signatures and genes under positive selection

#### Signatures of selection—the *d*_*i*_ approach

**Fig. 2:**
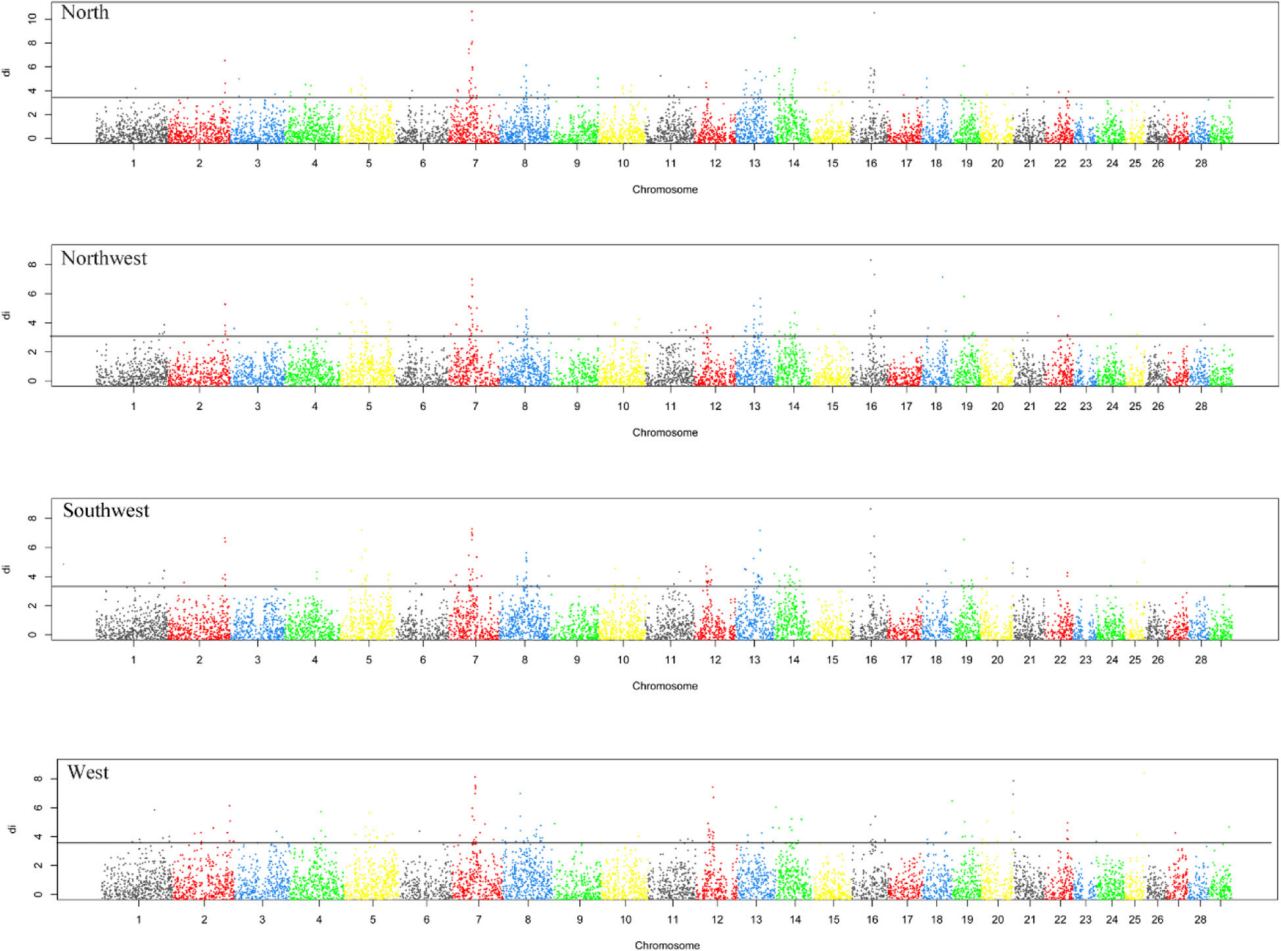
Genomic distribution of selection regions in four cattle groups (North, Northwest, Southwest and South). The distribution of average *di* value for each 50-SNP windows across auto chromosomes is plotted for each breed. Breeds are abbreviated as described in Additional file 1: Table S1

**Fig. 3:**
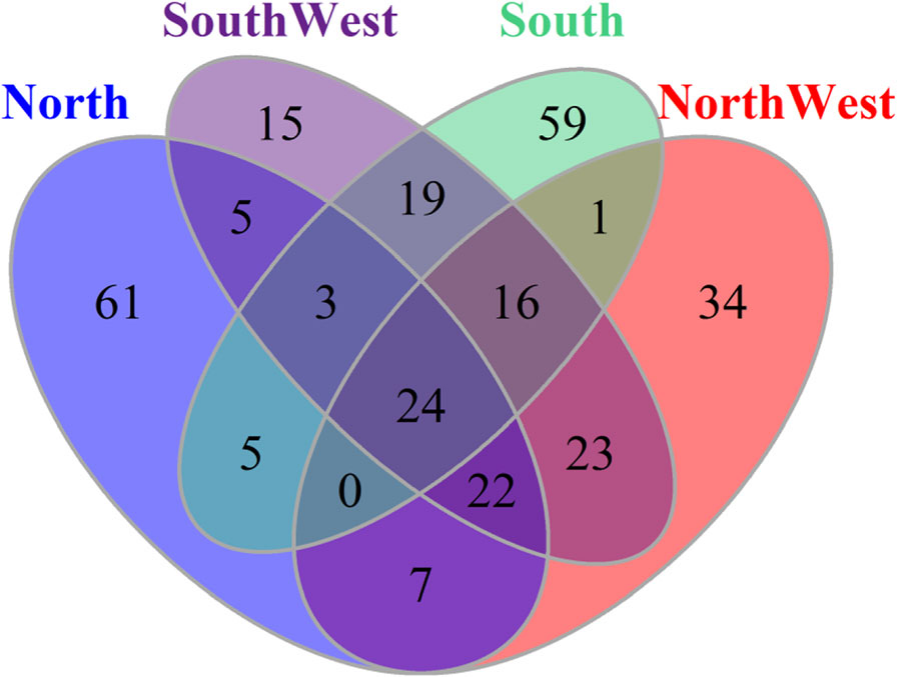
Venn diagram for shared versus group-specific selection events for top 1% (127) windows among four cattle groups (North, Northwest, Southwest and South).

Based on the results from MDS analysis, we divided eight populations into four groups: North (YHC and MGC), Northwest (CDM), Southwest (LSC, PWC and ZTC) and South (WSC and NDC). Totally, we identified the top 1% with highest average *di* values in the empirical distribution as candidate selection regions for each group. The genome-wide distribution of the standardized *di* values were generated for these groups (Fig. 2). We detected a total of 127 windows using top 1% criteria for each group. To identify shared and group-specific selection regions, we generated a Venn diagram based on these selection regions across four groups (Fig. 3), and estimated the numbers of overlapping regions among them. We obtained 294 candidate regions with 169 of them (~ 57.5%) uniquely detected in only one group (61 for North, 15 for Southwest, 34 for Northwest, and 59 for South). Moreover, we found 125 regions (~ 42.5%) were shared by two or more groups, 65 regions (~ 22.1%) were shared by three, and 24 (8.2%) regions were shared by four groups (Additional file 2: Table S2).

In this study, we identified 107, 103, 100, and 100 regions overlapping with genes in North, Northwest, Southwest and South group, respectively. Based on these regions, we totally detected 901 unique genes overlapped with the top 1% regions. Among them, there were 188, 71, 61 and 125 unique genes for each of North, Northwest, Southwest and South groups. We further performed DAVID analyses on identified genes in group-specific regions for each group. Notably, we found several functional clusters that were significantly enriched into functional importance processes among these breeds. In North group, we observed top three enriched clusters, including aromatic compound catabolic process, arylesterase activity, and response to toxic substance. In Northwest group, we discovered enrichments for ATP binding. In South group, we found most genes were enriched in lipid catabolic process, while no functional enrichment was observed in Southwest group.

In present study, we detected many candidate genes under positive selection which have been identified in previous reports, and three genes (*WIF1*, *HSPA9* and *UBE2D2*) were identified under positive selection in four groups (North, Northwest, Southwest and South) (see Table 1). Twelve genes were detected in more than two groups, including *RXFP2*, *LYN*, *TGS1*, *NPR2*, *SUOX, HBEGF, NMNAT1*, *SAR1B* and *DGAT1, RAB5B*, *PMEL* and *CDK2*. Notably, we observed several genes displayed group-specific selection which may potentially imply the adaption for local environments. For instance, *LCORL* was detected in the North group, four genes *PPP1R8*, *CPNE7*, *FANCA* and *SPG7* in the Northwest group, *BMP2* in Southwest group, and *LPGAT1* in the South group.

**Table 1:** Several selection regions and candidate genes with previous evidences across four groups including North, Northwest, Southwest and South groups

Chr	Start	End	Group	Candidate Gene	References
2	126,079,431	126,252,390	Northwest	*PPP1R8*	[34]
5	48,838,857	49,040,943	North; Northwest; Southwest; South	*WIF1*	[3]
5	57,490,826	57,729,851	Northwest; Southwest; South	*CDK2; DGKA; IKZF4 PMEL; RAB5B; RPS26; SUOX*	[36]
6	38,917,456	39,220,461	North	*LCORL*	[27]
7	4,529,302	4,762,339	Northwest; Southwest	*ELL, FKBP8, GDF15; ISYNA1 LRRC25; SSBP4*	[35]
7	47,559,314	47,821,784	North; Southwest	*SAR1B*	[30]
7	49,826,844	49,997,049	North; Northwest	*SPOCK1*	[3]
7	51,476,325	51,727,698	North; Northwest; Southwest; South	*HSPA9*	[30]
7	52,240,425	52,499,466	North; Northwest; Southwest; South	*UBE2D2*	[33]
7	53,107,671	53,401,992	North; Northwest; Southwest	*HBEGF*	[32]
8	60,334,817	60,589,007	Northwest; Southwest	*NPR2*	[4]
12	29,083,909	29,372,191	Southwest; South	*RXFP2*	[34]
13	49,457,188	49,707,937	Southwest	*BMP2*	[25]
14	1,549,928	1,901,893	Northwest; Southwest; South	*DGAT1*	[30]
14	24,769,617	25,001,051	Northwest; Southwest	*LYN; TGS1; RPS20*	[25]
16	44,291,131	44,636,778	North; Northwest; Southwest	*NMNAT1*	[30]
16	44,639,909	44,883,644	North; Northwest; Southwest	*PIK3CD; TMEM201*	[35]
16	73,481,886	73,711,818	South	*LPGAT1*	[28]
18	14,408,694	14,697,775	Northwest	*CPNE7; FANCA; SPG7*	[25]

#### Signatures of selection—the iHS approach

To explore the genomic regions under recent selection in diverse Chinese local cattle, we further carried out integrated haplotype score (iHS) analysis, because this approach could provide important insights into recent selections. We identified a total of 2315 (1263 unique) candidate regions across multiple chromosomes in eight breeds. Among them, we identified 294, 286, 285, 306,298, 289, 305 and 252 candidate regions in YHC, MGC, CDM, PWC, LSC, ZTC, WSC, and NDC cattle, respectively. Totally, we found 1425 genes under selection in 1263 nonredundant regions (Additional file 3: Table S3). Moreover, we found 548 unique genes under selection based on iHS values in two or more breeds, and 131, 120, 144, 98, 104, 88, 105, 87 were uniquely detected in YHC, MGC, CDM, PWC, LSC, ZTC, WSC and NDC, respectively. Additionally, we found some genes (e.g. lysozymes) were enriched in breakdown of pathogen’s cell wall macromolecules in MGC, while several genes were observed enriched in regulation of appetite and RNA phosphodiester bond hydrolysis in WSC and NDC, respectively.

#### Haplotype network analyses for candidate genes

**Fig. 4:**
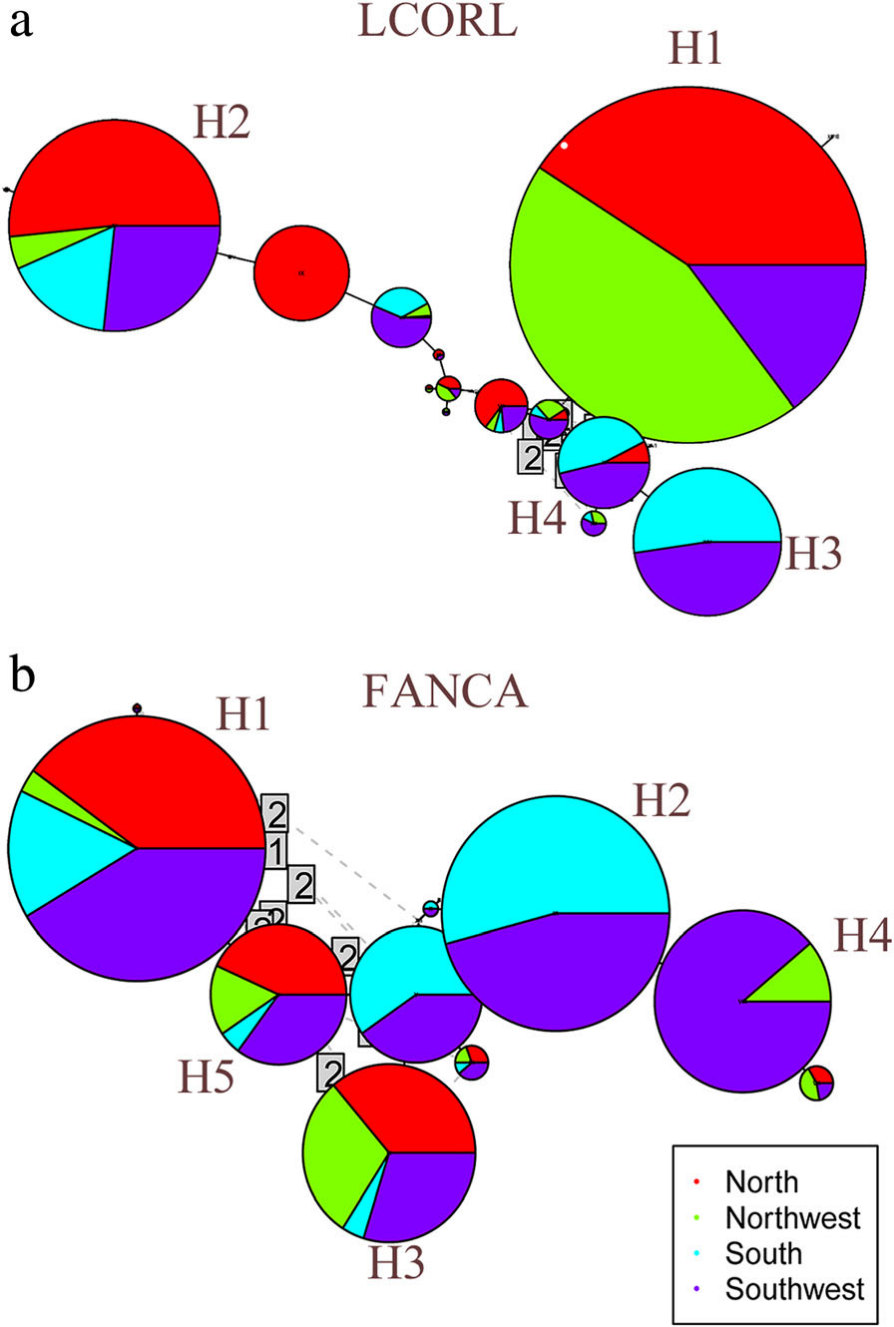
Analyses of haplotype networks for two genes. **a** *LCORL* and (**b**) *FANCA* for four cattle groups. Each node represents a different haplotype, with the size of the circle proportional to frequency. Circles are color coded according to group (red: North; blue: Northwest; cyan: Southwest; and green: South)

We further investigated the evolution history of several genes under selection by estimating haplotype diversity and constructing haplotype network. We found that the most common haplotype showed distinct frequency distribution among groups. Interestingly, we obtained 20 and 73 haplotypes within the 151 kb and 121 kb haploblock regions harboring *LCORL* and *LPGAT1*, and these two genes contained North and Northwest dominant haplotypes. Also, we found 23, 11 and 23 haplotypes within the 61 kb, 18 kb and 38 kb regions near *RXFP2*, *PPP1R8*, *FANCA*, and these genes had South and Southwest dominant haplotypes. For *LCORL*, the most common haplotype H1 (with frequency of 30.82%), was mainly found in North and Northwest cattle (YHC, MGC and CDM) and only minor portions occur in Southwest and South groups (PWC, LSC, ZTC, WSC and NDC) (Fig. 4a). H2 (with frequency of 18.40%) included a large proportion of Northern cattle (YHC and MGC). We also observed two haplotypes (H3 and H4) exclusive to indicine cattle (South and Southwest) with a frequency of (12.88 and 8.28%). Thus, this pattern may imply that separate haplotypes were clustered only for the indicine cattle (WSC and NDC), while other common haplotypes were identified for taurine cattle (YHC and MGC). PWC, LSC and ZTC were the only exceptions, as these breeds were derived from both taurine and indicine cattle. This was not unexpected, as it might mirror the complex ancestral backgrounds of breeds, i.e. these cattle are known as indicine breeds with taurine influences. For *FANCA*, we observed South and Southwest dominant haplotypes with high frequencies, H1 (21.39%) and H2 (19.22%), while three haplotypes (H1, H3, and H5) were shared by multiple groups from the North, Northwest, South and Southwest (Fig. 4b). We also found similar haplotype network pattern for *LPGAT1, PPP1R8* and *RXFP2* (Additional file 7: Figure S4).

## Discussion

We have performed population structure, admixture and phylogenetic analyses on diverse local cattle across a broad latitudinal range in China. Our analyses revealed the population structure was mostly correlated with the geographic distribution. These results were consistent with previous analyses using mitochondrial D-loop sequences and microsatellites in Chinese cattle, which also implied the geographical segregation of local breeds during breed formation [5, 7, 11]. Our findings also generally agreed with the history that the cattle from Northern and Southern China originate from two different sub-species *Bos t. taurus* and *Bos t. indicus* (zebu), followed by the recent introgression of European taurine cattle [9, 10]. In addition, we have shown that a certain degree of mixture among breeds from adjacent locations, suggesting the potential migration and introgression among them [54].

Based on population structure estimation, we conducted selection signature analyses for four groups in Chinese local cattle, including the North, Northwest, Southwest and South groups. To our knowledge, this is one of the first comprehensive studies on selection signatures using the high density genome-wide SNP array in Chinese local cattle. Our findings may offer valuable insights to understand the genetic mechanism of adaption in different local environmental conditions. In current study, on the group level, we used *di* to detect the locus-specific divergence derived from ancient selection events. On the other hand, on the breed level, we utilized iHS based on extend haplotype homozygosity to infer recent selection sweeps. An integrated strategy combining both could facilitate the detection of both ancient and recent selection events [55]. We totally detected 294 and 1263 candidate regions using the *di* and iHS approaches, respectively. When compared the identified regions from two methods, we found the 13.5 Mb overlapped regions, corresponding to 221 common genes. However, these two methods are targeting at the different levels (groups or breeds) at different time scales (ancient vs. recent events), therefore the overlaps do not necessarily imply the strongest selection signatures but may reflect the long-lasting selection events. Our combined data suggest that while Chinese cattle breeds share some selected regions, they also retain unique signatures of adaptation in responses to local environments.

Using the *di* method, we observed three genes (*WIF1*, *HSPA9* and *UBE2D2*) under selection in all four groups (North, Northwest, Southwest and South). *WIF1* and *HSPA9*, related to immune response, were implicated in previous investigations for selection evidences in multiple cattle populations [4, 23, 25, 30, 56]. *UBE2D2*, which may function as ligase activity and acid-amino acid ligase activity, has also been reported in East African Shorthorn Zebu [33]. Previous studies suggested common genomic regions across various populations may indicate historical selection shared between them, most likely due to ancestral (within archetypes), geographical similarities [30]. In present study, the identified shared genes may be associated with metabolic homeostasis or other common traits like disease resistance and behavior, and these shared selection genes may also reflect the pleiotropic effects of them on traits [57]. Several genes were detected in more than two groups, these genes include reduced bone mass and sexual maturation related genes (*RXFP2*) [19, 34, 58], stature related, birth and bone weight, and embryonic development related genes (*LYN*, *TGS1*, *NPR2*, *SUOX, HBEGF,* and *NMNAT1*) [4, 19, 21, 23, 30, 36, 59–63], milk production related genes (*SAR1B* and *DGAT1*) [4, 30, 31], and immune, coat colors and hypoxia adaptation related genes (*RAB5B*, *PMEL* and *CDK2*) [36, 64–67]. In addition, we found some candidate genes were previously reported in multiple species, for instance, genes for statue and development (*LCORL*, *NPR2*, and *BMP2*) and horns (*RXFP2*) have been identified in cattle, pig, horse and sheep, which could be due to parallel evolution because of similar nature and/or human-imposed selection [55].

Our study also revealed a list of genes within group-specific selection regions. These unique genes under selection can be responsible in shaping particular characteristics of the population, thus resulting in the origin and maintenance of that breed. For instance, in the North group, *LCORL* were identified as a candidate, and this gene has been previously reported in human, cattle, horse and pig and other domestic animals [4, 16, 18, 27, 68]. *LCORL* has been associated with height or stature in human genome-wide association study [68], as well as with body size in cattle [69], horses [70] and dogs [71]. As North group cattle normally have larger morphology and stature, this might be explained that local environment can influences body size with obvious correlations between cold and aridity, and body size [72, 73]. In the Northwest group, we detected four genes *PPP1R8*, *CPNE7*, *FANCA* and *SPG7* displaying groups-specific selection pattern which were related to reproduction, lipid metabolic, process cardiac system and nervous system development [25, 34, 74]. In the Southwest group, we found that *BMP2* gene showed selection signatures in cattle genome [4, 25, 55], and a previous study has suggested this gene may have critical role in controlling body dimension and muscle development [75]. In the South group, *LPGAT1* gene was identified as group-specific, which is supported by the previous evidence [28]. A recent study suggested that *LPGAT1* play an essential role in lipid synthesis in mice [76]. Also, *LPGAT1* may play a significant role in influencing BMI, percent body fat and hepatic triacylglycerol secretion in humans [77, 78]. Thus, based on the selection genes across genome, we suspected that cattle from Southwest group have been subjected for a longer time to tropical and humid environments and are well adapted to specific conditions. Additionally, these cattle may have interbred with more recently introduced European taurine individuals. These results generally agree with the recent findings from genomic analysis of climate adaptation in Mediterranean cattle breeds [73].

In addition, using the iHS approach, we totally found 1425 genes under selection in 1263 nonredundant regions (Additional file 3: Table S3). Moreover, 548 genes were identified in two or more breeds. Some genes have been previously described by previous studies in various populations. The iHS approach further identified several common genes within the shared regions that were most likely undergo similar selection pressure. For instance, we found *ZFHX4* and *LPCAT1* (related to milk production, somatic cell score and conformation) [79–81] in YHC and MGC within the North group. We detected *TNNI2, EPHX1* and *ROBO1* (associated with growth, meat quality and immune response, neuron differentiation) [35, 82–85] and *UBE2E1* and *GNA14* (related to hematological traits and high-altitude adaptation) in PWC, LSC and ZTC within Southwest groups, respectively [86, 87]. These genes can speculatively be interpreted as resulting from local adaption for mountain areas in the Southwest of China.

Notably, we observed numerous candidate genes displaying breed-specific selection in Chinese local cattle. For instance, we detected 28 candidate regions with the high iHS values on multiple chromosomes in the CDM breed from Northwest (Additional file 3: Table S3). These regions contained some functional important genes (*SLC16A7*, *PTPN4*, *NFE2L3*, *ENPP2*, *KITLG*, *CAPN2*, *KLHL29*, *ABHD15*, *FRMD4A*, *ALDH5A1*, and *AREG*), which are involved in growth, morphology, metabolism, meat quality and immune response. These identified genes have previously been suggested under selection in cattle or other farm animals [26, 29, 36, 60, 88–93]. Moreover, these selection genes detected in CMD may be important for physiological adaptations required to endure drought and hash in the areas of Caidamu Basin.

Our haplotype network analyses revealed a subset of candidate genes under selection. We found that common haplotypes were often present within the North and Northwest (taurine-derived) cattle, while separate distinct haplotypes were detected in the Southwest and South (indicine-derived) cattle, suggesting different evolutionary history for these populations. Also, haplotype diversity and their network pattern for positive selection genes, including *LPGAT1, LCORL, PPP1R8, RXFP2* and *FANCA*, also implied that these genes have been under different selection pressures in distinct environmental conditions. Continued efforts to sequence and analyze the genomes of local cattle populations, with a focus on under-sampled areas of the world, will provide a more comprehensive insight of genetic diversity, population structure and selection of diverse cattle population. Also, further attention is required to elucidate the mechanism underlying observed differences of morphological feature across populations under different environmental conditions [54, 73]. As many traits seem to be highly complex and may be influenced by many loci across the genome, it is probable that a considerable component of selection in cattle is polygenic and is yet to be discovered by exploring the genomic regions under selection using advanced statistics methods [94]. Also, new technology platforms like next generation sequencing may shed valuable insights for understanding the evolutionary history of cattle genome during the domestication process [95, 96]. In addition, the analysis bias caused by the genome assembly should be also been considered, and more accurate detection of selection signatures in diverse cattle population could be improved by analysis based on their own genome assemblies.

## Conclusions

Our results provided an important glimpse into diverse genomic selection during breed formation in Chinese local cattle. We identified a series of group-specific and breed-specific candidate genes, which are involved in immune response, sexual maturation, stature related, birth and bone weight, embryonic development, coat colors and adaptation. Furthermore, haplotype diversity and their network pattern for positive selection genes, including *LPGAT1, LCORL, PPP1R8, RXFP2* and *FANCA*, suggest that these genes have been under differential selection pressures in various environmental conditions.

## Additional files

Additional file 1: **Table S1**. The full names, associated abbreviation for each breeds and additional information on the locations of the sampling areas for eight populations. (XLSX 12 kb)

Additional file 2: **Table S2.** Candidate selective signature and genes under positive Selection using di approach for four groups including North, Northwest, South and Southwest. (XLSX 31 kb)

Additional file 3: **Table S3.** Candidate selective signature and genes under positive Selection using iHS approach for eight populations including YHC, MGC, CDM, PWC, LSC, ZTC, WSC and NDC. (XLSX 83 kb)

Additional file 4: **Figure S1.** The effective population sizes (Ne) of Chinese native cattle analyzed in this study. Ne of YHC, MGC, CDM, LSC, PWC, ZTC, WSC and NDC is plotted separately. X and Y axis represents generations and Ne respectively. (TIF 1662 kb)

Additional file 5: **Figure S2.** Neighbor-joining tree of eight populations. The tree was constructed using genetic sharing distances. (TIF 2514 kb)

Additional file 6: **Figure S3.** LD decay patterns in the eight breeds. Pairwise LD measures for all retained SNPs were performed using the PLINK “-ld” option. We used the default window size of 1 Mb, and set “–ld-window-r2” to 0 in order to get all pairs reported. The LD decay along genomic distance was fitted by smooth.spline function in R. (TIF 1673 kb)

Additional file 7: **Figure S4**. Haplotype networks of two loci. (A) LPGAT1 and (B) PPP1R8, (C) RXFP2 for four cattle groups. Each node represents a different haplotype, with the size of the circle proportional to frequency. Branch lengths are proportional to the number of nucleotide differences. Circles are color coded according to group (red: North, blue: Northwest, cyan: Southwest, and green: West). (TIF 1362 kb)

## Data Availability

The genotype data reported in this article are available upon request for research purpose.

## Abbreviations

BTA: *Bos Taurus* autosomes
CDM: Caidamu cattle
*Ho*: Observed heterozygosity
IBS: Identity-by-state
iHS: Integrated Haplotype Score
LD: Linkage disequilibrium
LSC: Liangshan cattle
MAF: Minor allele frequency
MDS: Multidimensional scaling
MGC: Menggu cattle
NDC: Nandan cattle
PWC: Pingwu cattle
QTL: Quantitative trait loci
SNP: Single nucleotide polymorphism
WSC: Wenshan cattle
YHC: Yanhuang cattle
ZTC: Zhaotong cattle
