# Supplementary figures and images for: Genome-wide scan reveals genetic divergence and diverse adaptive selection in Chinese local cattle
Source: BMC Genomics. 2019 Jun 14;20:494. doi: 10.1186/s12864-019-5822-y (PMC6570941; doi:10.1186/s12864-019-5822-y)

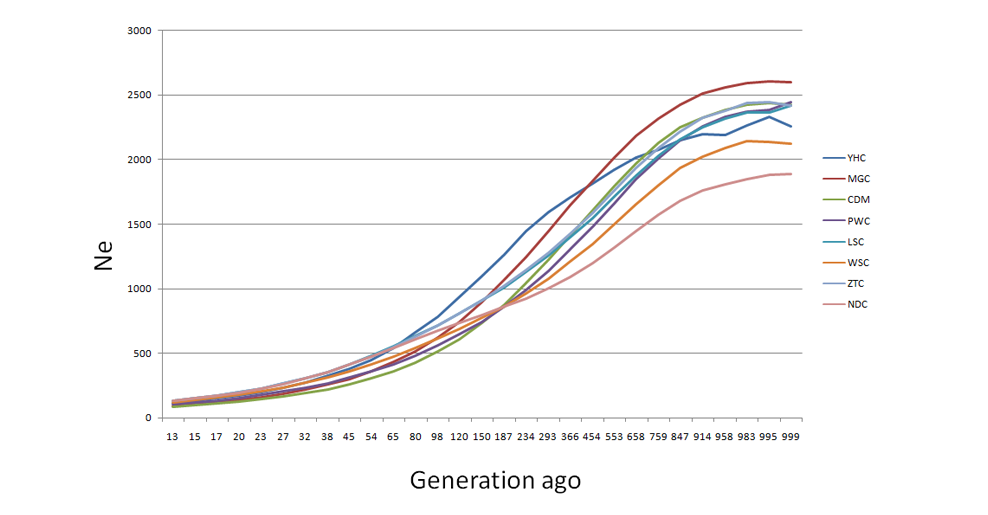

Supplement: Supplementary file 4 — Figure S1. The effective population sizes (Ne) of Chinese native cattle analyzed in this study. Ne of YHC, MGC, CDM, LSC, PWC, ZTC, WSC and NDC is plotted separately. X and Y axis represents generations and Ne respectively. (TIF 1662 kb) [file 12864_2019_5822_MOESM4_ESM.tif]

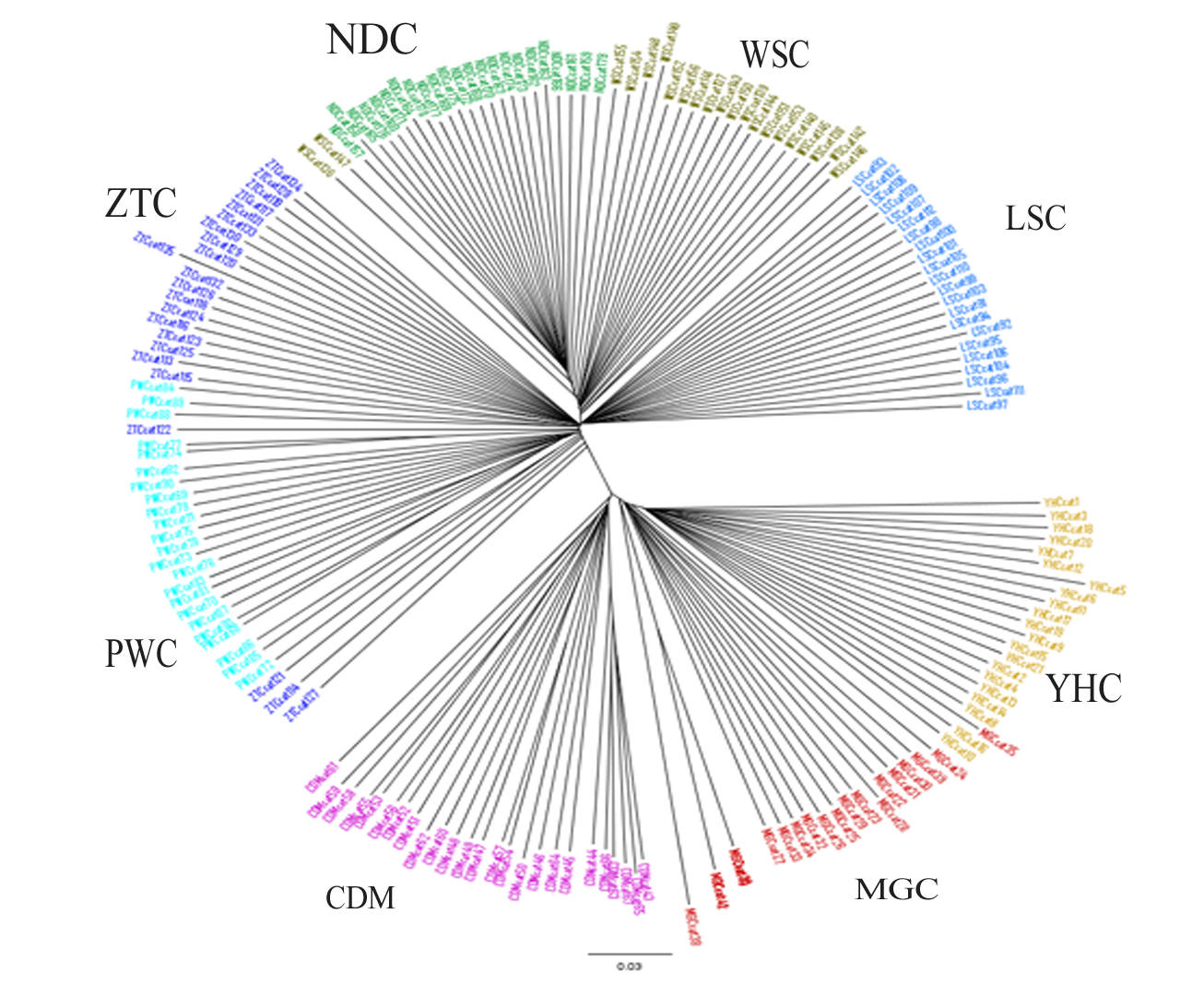

Supplement: Supplementary file 5 — Figure S2. Neighbor-joining tree of eight populations. The tree was constructed using genetic sharing distances. (TIF 2514 kb) [file 12864_2019_5822_MOESM5_ESM.tif]

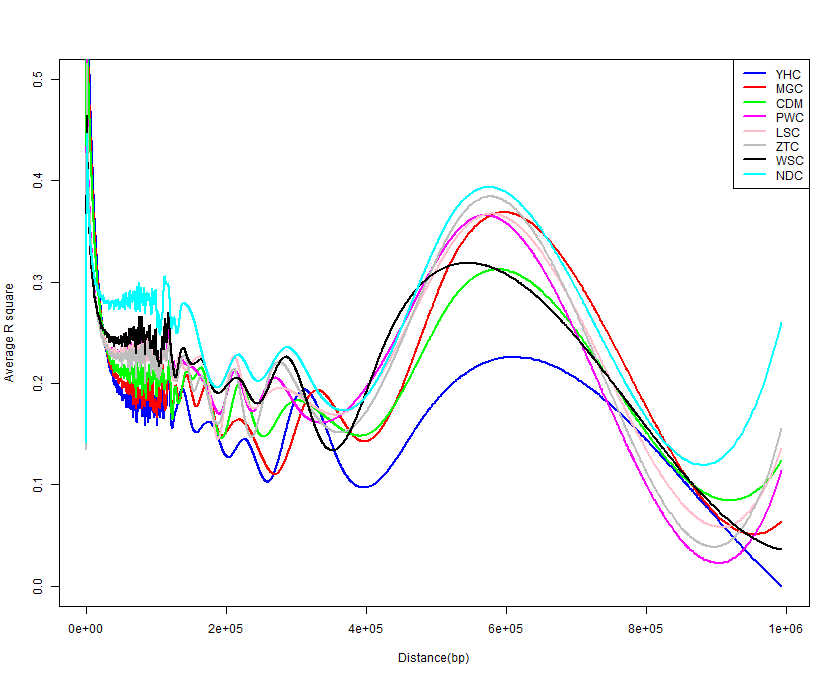

Supplement: Supplementary file 6 — Figure S3. LD decay patterns in the eight breeds. Pairwise LD measures for all retained SNPs were performed using the PLINK “-ld” option. We used the default window size of 1 Mb, and set “–ld-window-r2” to 0 in order to get all pairs reported. The LD decay along genomic distance was fitted by smooth.spline function in R. (TIF 1673 kb) [file 12864_2019_5822_MOESM6_ESM.tif]

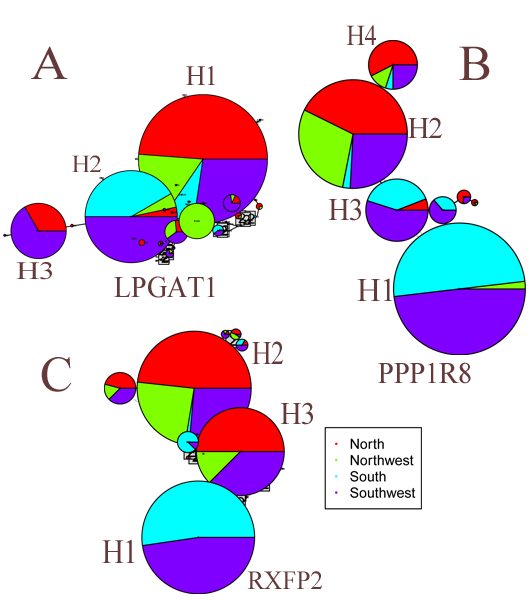

Supplement: Supplementary file 7 — Figure S4. Haplotype networks of two loci. (A) LPGAT1 and (B) PPP1R8, (C) RXFP2 for four cattle groups. Each node represents a different haplotype, with the size of the circle proportional to frequency. Branch lengths are proportional to the number of nucleotide differences. Circles are color coded according to group (red: North, blue: Northwest, cyan: Southwest, and green: West). (TIF 1362 kb) [file 12864_2019_5822_MOESM7_ESM.tif]
